# Are Neurophysiological Biomarkers Able to Discriminate Multiple Sclerosis Clinical Subtypes?

**DOI:** 10.3390/biomedicines10020231

**Published:** 2022-01-21

**Authors:** Daniele Belvisi, Matteo Tartaglia, Giovanna Borriello, Viola Baione, Sebastiano Giuseppe Crisafulli, Valeria Zuccoli, Giorgio Leodori, Antonio Ianniello, Gabriele Pasqua, Patrizia Pantano, Alfredo Berardelli, Carlo Pozzilli, Antonella Conte

**Affiliations:** 1 Department of Human Neurosciences, Sapienza University of Rome, 00185 Rome, Italy; daniele.belvisi@uniroma1.it (D.B.); matteo.tartaglia@uniroma1.it (M.T.); viola.baione@uniroma1.it (V.B.); sebastiano.crisafulli@uniroma1.it (S.G.C.); giorgio.leodori@uniroma1.it (G.L.); antonio.ianniello@uniroma1.it (A.I.); gabriele.pasqua@uniroma1.it (G.P.); patrizia.pantano@uniroma1.it (P.P.); alfredo.berardelli@uniroma1.it (A.B.); carlo.pozzilli@uniroma1.it (C.P.); 2 Istituto di Ricerca e Cura a Carattere Scientifico (IRCCS) Neuromed, 86077 Pozzilli, Italy; 3 MS Center Neurology Unit, San Pietro Fatebenefratelli Hospital, 00189 Rome, Italy; borriello.giovanna@fbfrm.it; 4 Department of Statistical Sciences, University of Padua, 35121 Padua, Italy; valeria.zuccoli.ext@alumni.unipd.it; 5 Multiple Sclerosis Center, S. Andrea Hospital, Department of Neurology and Psychiatry, Sapienza University of Rome, 00185 Rome, Italy

**Keywords:** multiple sclerosis, disease progression, neurophysiology, biomarkers, transcranial magnetic stimulation, grey matter inhibitory mechanisms

## Abstract

Secondary progressive multiple sclerosis (SPMS) subtype is retrospectively diagnosed, and biomarkers of the SPMS are not available. We aimed to identify possible neurophysiological markers exploring grey matter structures that could be used in clinical practice to better identify SPMS. Fifty-five people with MS and 31 healthy controls underwent a transcranial magnetic stimulation protocol to test intracortical interneuron excitability in the primary motor cortex and somatosensory temporal discrimination threshold (STDT) to test sensory function encoded in cortical and deep grey matter nuclei. A logistic regression model was used to identify a combined neurophysiological index associated with the SP subtype. We observed that short intracortical inhibition (SICI) and STDT were the only variables that differentiated the RR from the SP subtype. The logistic regression model provided a formula to compute the probability of a subject being assigned to an SP subtype based on age and combined SICI and STDT values. While only STDT correlated with disability level at baseline evaluation, both SICI and STDT were associated with disability at follow-up. SICI and STDT abnormalities reflect age-dependent grey matter neurodegenerative processes that likely play a role in SPMS pathophysiology and may represent easily accessible neurophysiological biomarkers for the SPMS subtype.

## 1. Introduction

Multiple sclerosis (MS) is a chronic inflammatory and demyelinating disease of the central nervous system (CNS) and is one of the leading causes of disability in young adults [1]. MS is clinically characterized by different phases and clinical subtypes, with relapsing-remitting multiple sclerosis (RRMS) being the most common clinical variant. RRMS is characterized by relapses of acute neurological symptoms that end with partial or complete remission [2]. During the course of the disease, people with MS (pwMS) may show a gradual and irreversible worsening of neurological disability that can emerge as progression from RRMS to secondary progressive multiple sclerosis (SPMS) [3]. Early identification of the SPMS subtype is particularly challenging since SPMS conversion is characterized by a subtle onset, thus implying clinical and retrospective identification with a significant diagnostic delay [3,4]. Unlike RRMS, which mostly involves pathophysiological mechanisms of white matter inflammation and demyelination, a growing body of evidence has reported that progression from RRMS to SPMS is a consequence of grey matter atrophy at the cortical and subcortical level [5,6,7].

Somatosensory evoked potentials and motor potentials evoked by transcranial motor cortex stimulation reveal demyelinating damage and reduced conduction velocity in the central sensory and motor pathways in early stages of RRMS, but they are less effective in demonstrating changes reflecting grey matter loss [8,9]. However, other neurophysiological techniques can test motor and sensory circuits at the cortical and subcortical grey matter level. Of the transcranial magnetic stimulation (TMS) techniques testing motor circuits, short-interval intracortical inhibition (SICI) and intracortical facilitation (ICF) can be used to assess intracortical inhibitory and excitatory interneuron activity in the primary motor cortex (M1) [10,11] and to test the integrity of M1 cortical grey matter [10,11]. In regard to neurophysiological assessment of sensory function, somatosensory temporal discrimination threshold (STDT) is an easily accessible technique to test the temporal processing of tactile stimuli [12,13,14,15,16]. Studies testing STDT in pwMS have reported abnormally increased STDT values [15,17,18]. It was initially hypothesized that white matter damage may contribute to this abnormality [15], but more recent neurophysiological and neuroimaging investigations have clearly demonstrated that increased STDT values reflect grey rather than white matter damage in pwMS [17,18].

The aim of the present study was to verify whether TMS parameters and STDT measures of motor and sensory grey matter circuits at the cortical and subcortical level are associated with the SPMS rather than RRMS subtype. To do so, we tested: (1) M1 excitability and intracortical inhibitory and facilitatory circuits using single- and paired-pulse TMS paradigms [19] and (2) S1 and deep grey matter neural structures involved in the temporal discrimination of sensory stimuli using STDT [13,20,21,22]. Finally, to investigate whether the neurophysiological measures at baseline also predicted disability in both RRMS and SPMS, we clinically re-assessed pwMS at least one year after the first neurophysiological evaluation and investigated possible associations between neurophysiological variables at baseline and disability scale scores at follow-up.

## 2. Materials and Methods

### 2.1. Participants

Fifty-five pwMS (37 RRMS, 18 SPMS) and 31 age- and sex-matched healthy controls (HCs) were enrolled (Table 1, Table 2 and Table S1). PwMS were consecutively enrolled during their regular outpatient clinic visits. HCs were consecutively enrolled from a pool of healthy volunteers. MS diagnosis was defined according to the 2017 revised McDonald criteria [23]. Clinical subtype was assigned by neurologist experts in MS [24,25]. Neurophysiologist investigators were blinded to the clinical evaluation. The inclusion criteria were age over 18 years, clinical stability, no corticosteroid intake in the 30 days preceding the assessment, and a Mini-Mental State Examination (MMSE) score >26 for STDT testing [26]. Exclusion criteria were contraindications to TMS (i.e., history of epilepsy, pacemaker implantation, head trauma). Subjects with a clinical history or clinical signs of sensory polyneuropathy and carpal tunnel syndrome were excluded. PwMS continued their usual disease-modifying therapy and domiciliary therapy. PwMS were only asked to undergo a 24 h withdrawal from symptomatic medications. Each participant underwent clinical and neuropsychological examinations, including the Expanded Disability Status Scale (EDSS) [27] and MMSE. The Charlson comorbidity index was used to measure comorbities burden in RRMS and SPMS individuals. All pwMS were clinically re-evaluated one year after baseline assessment. The study was conducted in accordance with the Declaration of Helsinki and approved by the ethics committee of Sapienza University of Rome. Written informed consent was obtained from all participants (protocol number 4570).

### 2.2. Neurophysiological Assessment

#### 2.2.1. Paired-Pulse TMS to Test M1 Intracortical Interneuron Excitability

**Figure 1:**
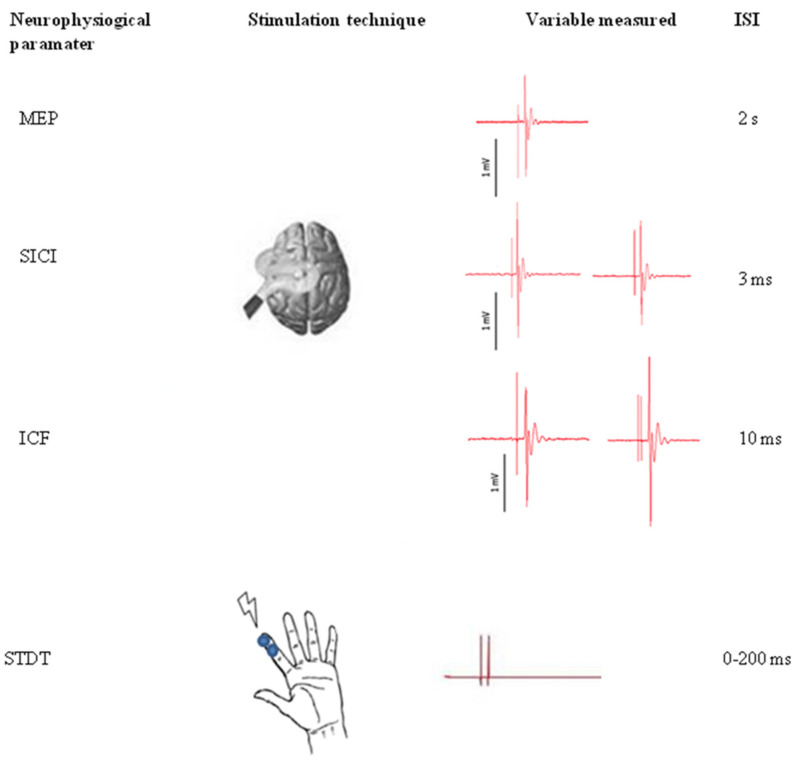
Experimental paradigm. Transcranial magnetic assessment: To elicit motor evoked potentials from the contralateral first dorsal interosseous muscle, single- and paired-pulse transcranic magnetic stimulation (TMS) were delivered on the motor area of the right hand. Paired-pulse TMS was delivered using an interstimulus interval (ISI) of 3 ms (short intracortical inhibition, SICI) and 10 ms (intracortical facilitation, ICF). Somatosensory temporal discrimination threshold: pairs of electric stimuli were delivered on the volar skin of the right index finger. Starting from an ISI of 0 ms (simultaneous stimuli), 10 ms steps were performed until participants were able to recognize the two stimuli as temporally separate.

Single- and paired-pulse TMS were delivered using a Magstim Bistim2 magnetic stimulator (The Magstim Company, Ltd., Whitland, South West Wales, UK). Magnetic pulses were delivered through a figure-of-eight coil placed on the M1 motor area of the right hand. The coil was placed tangentially to the scalp, and the tail was directed backward and at 45° to the median line to elicit MEPs from the contralateral first dorsal interosseous (FDI) muscle. Resting motor threshold (RMT) was defined as the minimum intensity able to evoke MEPs of at least 50 µV of amplitude in at least 5 of 10 consecutive trials. Single-pulse intensity was set to obtain MEPs of 1 mV of average amplitude [28]. Paired-pulse TMS was delivered using an ISI of 3 ms (SICI) and 10 ms (ICF). Conditioning stimulus intensity was fixed at 80% of RMT, and test stimulus intensity was the same as that used to obtain 1 mV MEPs. At least 20 trials for each ISI were recorded. SICI and ICF effects were calculated as the conditioned MEP amplitude percentage with respect to the test MEP amplitude (SICI (%) and ICF (%)). Figure 1 shows the experimental paradigm.

#### 2.2.2. STDT to Test S1 Intracortical and Deep Grey Matter Nuclei Interneuron Excitability

With subjects seated comfortably on an armchair in front of a table, STDT was assessed following experimental procedures used in previous studies [29,30,31,32]. We delivered pairs of electric stimuli starting from an ISI of 0 ms (simultaneous stimuli), which was increased in 10 ms steps. Stimulation consisted of square-wave electrical pulses delivered by AgCl electrodes placed on the volar skin of the right index finger (anode was located 0.5 cm distally from the cathode) and connected to an electric stimulator (Digitimer DS7AH). Stimulation intensity was defined as the minimum intensity at which the participant could perceive 10 out of 10 consecutive stimuli. To assess stimulation intensity, stimuli were delivered starting from 2 mA and increasing by 1 mA for each step. STDT was considered as the first of three consecutive ISIs at which participants recognized stimulus pairs as temporally separate. To maintain attention level and minimize the risk of a perseverative response during STDT assessment, some “catch trials” were included, which involved the random delivery of pairs of simultaneous stimuli. Neurophysiological techniques were performed in a pseudo-randomized order.

### 2.3. Statistical Analysis

Statistical analysis was performed using R, version 4.0.2.

T-test: An unpaired *t*-test was applied to all variables to compare pwMS and HCs. Variances were assumed to be unknown and to differ between groups, so Welch correction was applied to the statistic.

Logistic regression and validation: Logistic regression was used to discriminate SPMS from RRMS pwMS. For computational purposes, pwMS with RRMS were coded as class 0, while an SPMS diagnosis was coded as class 1. EDSS was removed from the list of independent variables in order to focus on neurophysiological parameters. Meanwhile, age and disease duration were clinically considered correction factors for other variables. However, age was chosen since it showed higher between-group differences than disease duration. Firstly, all explanatory variables were corrected for age as a multiplicative effect and an automatic stepwise logistic regression was performed to select the most important variables. This procedure was driven by Akaike Information Criterion (AIC), starting from the empty model, i.e., considering only the intercept. At each step, the system included or excluded a single variable depending on how much this change would have lowered the AIC model. The sequence of steps was interrupted when none of the possible model changes would have caused a decrease in AIC. At the end of this automatic variable selection, only significant variables were kept (*p* < 0.05), and the final model was estimated. Finally, model predictions were computed and validated with leave-one-out cross-validation.

Correlation: Explanatory variables were tested for mutual correlation by computing Spearman’s coefficient of rank correlation. Pairwise complete observations were used for each pair of variables. This approach exploited all data, was not significantly impacted by missing values, and did not require imputation. For each correlation coefficient, its raw *p* value was calculated. An adjusted *p* value was computed using the Bonferroni method to correct for multiple comparisons.

Longitudinal assessment: To explore the relationship between neurophysiological parameters and disability progression, we designed a linear regression model with the follow-up EDSS score as the dependent variable and the neurophysiological parameters that best discriminated between subtypes at baseline as independent variables.

## 3. Results

Single- and paired-pulse TMS assessment showed that pwMS had a significantly lower SICI than HCs. Conversely, MEP amplitude and ICF were similar in pwMS and HCs. STDT values significantly differed between the groups, being higher in pwMS than in HCs (Table 3).

### 3.1. Logistic Regression Model

**Figure 2:**
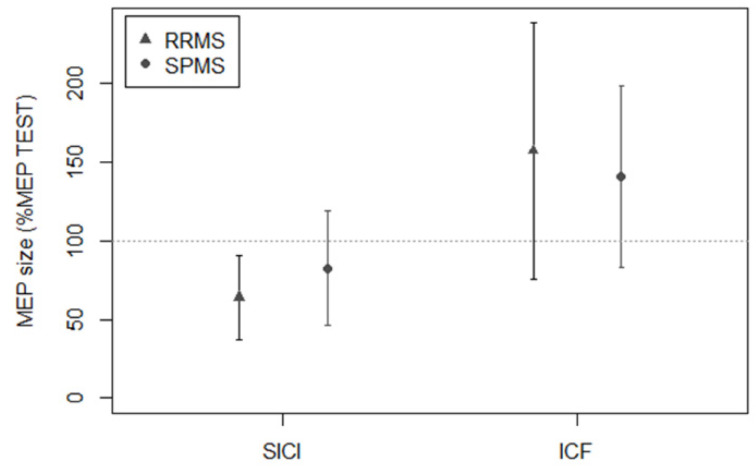
Transcranial magnetic stimulation assessment in subjects with RRMS and SPMS. SICI, as tested by paired-pulse stimulation with a 3 ms ISI, was significantly lower in SPMS than in RRMS. ICF, as tested by paired-pulse stimulation with a 10 ms ISI, was similar in RRMS and SPMS. Y axis: MEP size, expressed as conditioned MEP amplitude/test MEP amplitude × 100. Bars represent standard deviation.

**Figure 3:**
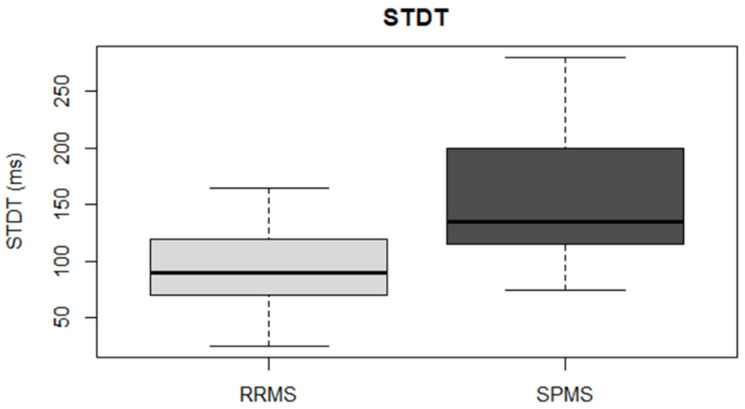
Somatosensory temporal discrimination threshold. SPMS showed significantly higher STDT values than RRMS.

RRMS and SPMS differed in terms of age (RRMS: 42.19 ± 8.38 years; SPMS: 51.44 ± 7.63 years; *p* = 2.29 × 10^−4^), disease duration (RRMS: 11.11 ± 8.05 years; SPMS: 17.06 ± 9.88 years; *p* = 0.03), and baseline EDSS score (RRMS: 2, IQR: 1–3; SPMS: 5, IQR: 4.5–6; *p* = 2.34 × 10^−9^). Conversely, sex distribution was similar in the two groups (RRMS: 64% females; SPMS: 50% females; *p* = 0.3). For logistic regression analysis, subjects with RRMS were coded as class 0, while subjects with SPMS were coded as class 1. Logistic regression analysis showed that SICI and STDT were the neurophysiological parameters of the best estimated model (data shown in Table 4 and Figure 2 and Figure 3). Model predictions were estimated and validated with leave-one-out cross-validation. The evaluation metrics were: accuracy, 0.836; sensitivity, 0.777; and specificity, 0.865. Formula (1), obtained by the logistic regression model to compute the probability that a subject (*X*) would be assessed as SPMS (class 1), was the following:

(1) $$P\left(X=1\right)=\frac{e^{-5.95503+0.00056*\mathrm{SICI}\left(\%\right)*\mathrm{Age}+0.00073*\mathrm{STDT}*\mathrm{Age}}}{1+e^{-5.95503+0.00056*\mathrm{SICI}\left(\%\right)*\mathrm{Age}+0.00073*\mathrm{STDT}*\mathrm{Age}}}$$

ROC curve was used to test the sensitivity and specificity of SICI and STDT separately. For SICI, the evaluation metrics were: area under the curve (AUC), 0.65; sensitivity, 0.588; and specificity, 0.679. For STDT, the evaluation metrics were: AUC, 0.805; sensitivity, 0.944; and specificity, 0.543.

### 3.2. Cross-Sectional and Longitudinal Clinical Correlates of SICI and STDT in People with MS

**Figure 4:**
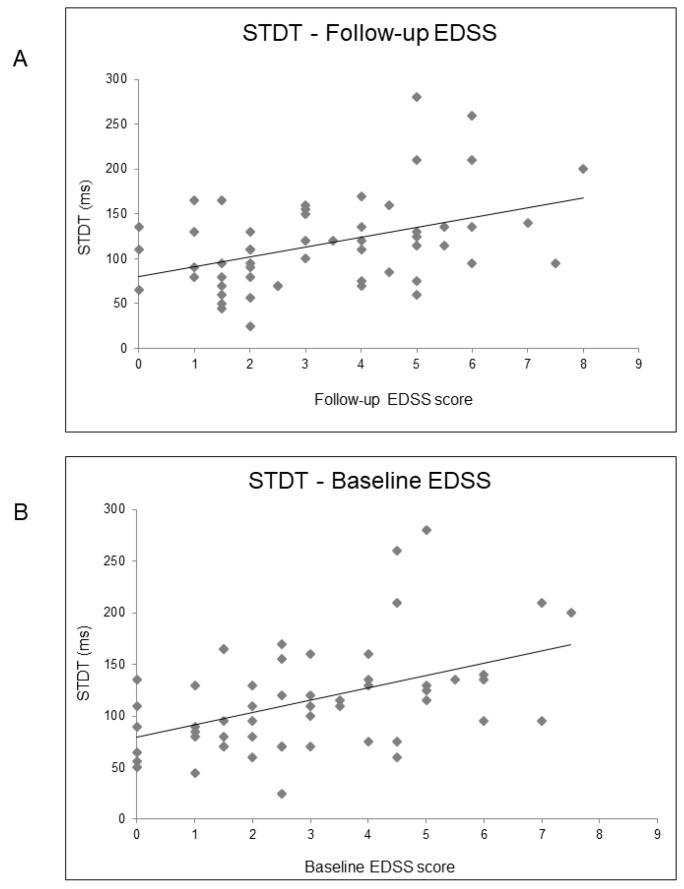
Relationship between STDT values and EDSS score at follow-up (Panel **A**) and EDSS score at baseline (Panel **B**).

In order to identify possible clinical correlates of SICI and STDT in pwMS, we performed an age-corrected partial Spearman’s rank correlation coefficient analysis, which revealed a correlation between STDT values and disease duration (rho = 0.34; *p* = 0.02) and EDSS score (rho = 0.45; *p* = 0.001) (Figure 4). However, SICI did not correlate with clinical variables. In order to investigate whether STDT abnormalities may depend on sensory disturbances, we explored possible correlations between STDT values and EDSS sensory domain scores in RRMS and SPMS subjects. We found no correlations in either group (RRMS: rho = 0.29; *p* = 0.1; SPMS: rho = 0.15; *p* = 0.5).

Finally, we performed a one-year follow-up clinical assessment and observed an increase in EDSS score (*p* = 0.006). We designed a linear regression model with the follow-up EDSS score as the dependent variable and SICI, STDT, age, and disease duration as independent variables. The model also showed that SICI (B = 0.021; t = 2.69; *p* = 0.01), STDT (B = 0.013; t = 2.86; *p* = 0.007) (Figure 4), and age (B = 0.112; t = 3.75; *p* = 0.001) were associated with the follow-up EDSS score. The Charlson comorbidity index was similar in RRMS and SPMS and did not correlate with neurophysiological parameters.

## 4. Discussion

In the present study, we evaluated possible neurophysiological biomarkers for MS subtypes by testing the activity of neural circuits of both motor and sensory grey matter cortical and subcortical structures. Consistent with the hypothesis that grey matter damage is involved in MS progression [6], we observed that STDT and SICI abnormalities, which both reflect grey matter interneuron activity [10,11,17], were significantly associated with the SPMS subtype. We thus provided a mathematical model to compute the probability that a pwMS would be assessed as the SPMS subtype by combining SICI and STDT values. Finally, we observed that both SICI and STDT values at baseline were associated with EDSS scores at follow-up, thus suggesting that the neurophysiological biomarkers we tested at baseline may also potentially predict disability progression.

During the experimental procedures, we took several precautions to minimize possible confounding factors due to methodological biases. Investigators performing the neurophysiological evaluation were blinded to pwMS clinical features. To avoid our findings being affected by fatigue mechanisms, learning processes, or attention level, all techniques were performed in a pseudo-randomized order. Since TMS responses, including SICI and ICF, are influenced by target muscle voluntary contraction [33], we asked pwMS to relax and monitored background EMG activity continuously during data collection. Trials showing EMG background activity greater than 100 μV in the 100 ms preceding magnetic pulses were rejected. In addition, we performed “catch trials” consisting of a single stimulus delivered randomly to check for changes in the participant’s attention during STDT assessment [34]. In order to verify whether a comorbidities burden could have affected our findings, we calculated the Charlson comorbidity index. It showed that the weight of comorbid conditions did not differ in RRMS and SPMS individuals and did not correlate with neurophysiological parameters.

A growing body of evidence has reported that cortical and subcortical grey matter atrophy, rather than white matter damage, is involved in the pathophysiology of SPMS [5,6,7]. Histology studies have demonstrated that imaging-derived grey matter atrophy in subjects with SPMS reflects neurodegeneration [35] with an extensive loss of synapses and a focal loss of neuronal bodies in the cortex and deep grey matter regions, possibly leading to a loss of brain capacity to adapt to tissue damage [36,37]. SICI and STDT both reflect grey-matter-mediated inhibitory mechanisms that involve cortical (M1 for SICI and S1 for STDT) and subcortical circuits (STDT) [10,11,17]. The significant association we observed between SICI and STDT abnormalities and SPMS suggests that the loss of inhibition in cortical and deep grey matter nuclei may play a key role in SPMS.

Our finding of an association between SPMS and SICI, but not ICF, deserves a comment. Although ICF, like SICI, is elicited by a paired-pulse TMS protocol, it recruits excitatory intracortical pathways in M1 with glutamatergic mediation [10,11]. The different propensity we observed between SICI and ICF in their association with SPMS may suggest that neurodegenerative processes involved in MS progression and grey matter atrophy induce more pronounced alterations in inhibitory circuits (of both motor and sensory systems), rather than non-specific detrimental effects on grey matter circuitry. Alternatively, it is not the amount but the time course of the appearance of abnormalities that explains the different association of SICI and ICF with SPMS, i.e., earlier inhibitory circuit dysfunction and later excitatory circuit dysfunction. In line with this hypothesis, glutamate levels in the brain have been found to be increased in the normal-appearing white matter of all MS subtypes as compared with HCs [38], potentially due to increased production by inflammatory cells in combination with reduced clearance by oligodendrocytes and astrocytes [39], whereas later, there is evidence of a continuous glutamate decline [40].

Previous studies in healthy subjects showed that age influences inhibitory-mediated mechanisms in motor [41] and sensory functions [42,43,44]. The mathematical model derived from our regression analysis also includes age as a factor in the formula predicting SPMS. Both aforementioned clinical and neurophysiological results are consistent with our findings and strengthen our hypothesis that SICI and STDT reflect age-dependent inhibitory mechanisms involved in SPMS.

The follow-up clinical assessment we performed allowed us to explore the clinical weight of SICI and STDT by investigating neurophysiological–clinical correlations. We found that STDT correlated with both disease duration and disability level, as tested by EDSS scores at baseline and follow-up, whereas SICI did not correlate with clinical variables at baseline but was associated with EDSS score at follow-up. As mentioned above, SICI and STDT both depend on cortical activity, whereas subcortical structures are involved only in STDT generation, with the thalamus playing a crucial role [17]. Studies investigating the spread of grey matter damage in pwMS revealed that the thalamus is involved from the earliest stages. The thalamus is one of the first regions to become atrophic in MS [45,46,47], and the rate of thalamic atrophy remains high throughout the disease course [46]. In addition, thalamic atrophy correlates with disease duration and disability accumulation in MS [46]. It is therefore plausible that the clinical correlations of STDT here observed reflect the ability of STDT to detect thalamic atrophy progression in MS. Concordantly, a recent study found a significant correlation between STDT values and thalamic volume and, a four-year follow-up investigation found that STDT increased over time in pwMS but not in HCs [31], thus supporting our findings that STDT may contribute to a marker of disease progression in MS. The observation that SICI was associated with EDSS score at follow-up, which was significantly increased with respect to baseline, suggests that SICI may be a predictor of disease trajectory. From a pathophysiological point of view, SICI may reflect the progressive involvement of cortical grey matter during the disease course.

The present study has a number of limitations. First, since we aimed to provide neurophysiological markers that could be applied in routine clinical practice, we enrolled pwMS who were on different symptomatic and disease-modifying therapies. Although this approach reflected the real-world condition of pwMS, it is possible that the presence of medication may have at least partly influenced our results. Although we asked pwMS to undergo a 24 h withdrawal from symptomatic medications, we cannot fully exclude long-term effects. Second, our study had a cross-sectional design and only one year of follow-up. Although EDSS scores were significantly increased at follow-up, thus implying disease progression in at least some of the pwMS we tested, the relationships between neurophysiological parameters and SPMS need to be validated by longitudinal studies with a longer follow-up. In addition, in the present study, we did not specifically assess the possible contribution of fatigue on the neurophysiological measures we tested. Therefore, future studies investigating the relationship between fatigue and grey matter involvement in MS are needed.

Finally, in the present study, we did not perform a conventional or advanced magnetic resonance imaging (MRI) assessment, which could have provided further support to the current hypothesis regarding the relationship between SICI and STDT abnormalities and grey matter damage in MS. Conventional MRI techniques are able to detect only 20–30% of cortical lesions and do not discriminate between subtypes [5]. Conversely, advanced MRI techniques investigating functional connectivity and grey matter network patterns have recently shown a higher ability to predict SPMS transition [6], but this approach entails high cost and experienced staff. The correlation between neurophysiological measures and advanced MRI techniques deserves investigation in future studies.

The observation that abnormalities in two neurophysiological parameters reflecting cortical and subcortical grey matter inhibitory mechanisms of the motor and sensory systems were associated with SPMS suggests that neurodegeneration, particularly loss of inhibition, plays a key role in SPMS. We conclude that SICI and STDT are easily accessible pathophysiological candidate biomarkers that may increase the accuracy and timeliness of SPMS identification. Future multicenter longitudinal studies are needed to verify our proposed neurophysiological index.

## Figures and Tables

**Table 1:** Demographic and clinical features of people with multiple sclerosis and healthy controls.

	pwMS	Healthy Controls	*p* Value
Age	45.2 ± 9.2 years	41.1 ± 6.9 years	0.521
Sex, n	F: 33 M: 22	F: 16 M: 15	0.461
Age at onset	32.1 ± 8.7 years	-	
Disease duration	13.1 ± 9.1 years	-	
Baseline EDSS score	2.5 (range: 1.5–4.5)	-	
Follow-up EDSS score	3.0 (range: 1.5–5.0)	-	

EDSS: expanded disability status scale; pwMS: people with MS. Data are expressed as mean values ± SD except for EDSS expressed as median values.

**Table 2:** Relapsing–remitting and secondary progressive patients’ clinical and radiological features at baseline.

	RR	SP
EDSS score	2.0 (range: 1.0–3.0)	5.0 (range: 4.5–6.0)
9HPT dominant hand (s)	22 ± 3.2	30.4 ± 9.5
9HPT nondominant hand (s)	22.1 ± 2.9	31 ± 10.2
T25FW (s)	5.9 ± 1.5	9.3 ± 2.5
Brain lesion load (mL)	9.926 ± 8.555	17.816 ± 11.850

9HPT: 9-hole peg test, EDSS: expanded disability status scale, RR: relapsing-remitting, SP: secondary progressive, T25FW: timed 25-foot walk. Data are expressed as mean values ± SD except for EDSS expressed as median values.

**Table 3:** Neurophysiological parameters of people with multiple sclerosis and healthy controls.

	pwMS Mean Values	HC Mean Values	*p* Value
MEP	0.95 ± 0.85	1.09 ± 0.23	0.287
SICI (%)	71.02 ± 31.65	27.33 ± 13.21	2.00 × 10^−10^
ICF (%)	151.26 ± 73.47	184.32 ± 51.55	0.051
STDT	114.83 ± 51.99	53.20 ± 20.17	4.95 × 10^−11^

HC: healthy control; MEP: motor evoked potential; SICI: short intracortical inhibition; ICF: intracortical facilitation; pwMS: people with multiple sclerosis; STDT: somatosensory temporal discrimination threshold.

**Table 4:** Logistic regression model. Dependent variable: multiple sclerosis subtype (RRMS vs. SPMS).

	Estimate	*p* Value	Odds Ratio	95% Confidence Interval
Intercept	−5.95503	1.53 × 10^−5^	0.00259	−9.09252–3.58799
SICI (%) * age	0.00056	1.84 × 10^−4^	1.00056	1.00013–1.00114
STDT * age	0.00073	2.71 × 10^−2^	1.00073	1.00040–1.00117

SICI: short intracortical inhibition; STDT: somatosensory temporal discrimination threshold; *: corrected for.

## Data Availability

The data presented in this study are available on request from the corresponding author.

## Supplementary Materials

The following are available online at https://www.mdpi.com/article/10.3390/biomedicines10020231/s1, Table S1: Patient’s demographic, clinical and neuroimaging characteristics.
