# Supplementary material for: Are Neurophysiological Biomarkers Able to Discriminate Multiple Sclerosis Clinical Subtypes?
Source: Biomedicines. 2022 Jan 21;10(2):231. doi: 10.3390/biomedicines10020231 (PMC8869727; doi:10.3390/biomedicines10020231)
Supplement: Supplementary file 1 [file biomedicines-10-00231-s001.zip › biomedicines-1515609-supplementary.pdf]

Table S1. Patient's demographic, clinical and neuroimaging characteristics. 9HPT: 9-hole peg test; ANA: antinuclear antibody; BoNT: botulinum toxin; BZD: benzodiazepine; Delta-9-THC: delta-9-tetrahydrocannabinol; DD: disease duration; DMD: disease modifying drug; EDSS: expanded disability status scale; F: female; M: male; N/A: not applicable; RR: relapsing-remitting; SNRI: serotonin–norepinephrine reuptake inhibitor; SP: secondary progressive; SSRI: selective serotonin reuptake inhibitors; T25FW: timed 25-foot walk.

ID	Age (y)	Sex	Clinical Phenotype	DD (y)	EDSS	DMD	Symptomatic Therapy	Comorbidities	9HPT Dominant Hand (s)	9HPT Nondominant Hand (s)	T25FW (s)	Brain Lesion Load (mL)	Spinal Lesion
1	42	F	RR	13	3.0	Alemtuzumab	None	None	21.5	22.5	5.2	21.530	Yes
2	42	F	RR	5	1.0	Dimethyl fumarate	None	Thyroiditis	21.0	22.5	5.5	13.537	No
3	27	M	RR	11	3.0	Fingolimod	Baclofen, Pregabalin	None	23.3	24.0	5.8	3.833	Yes
4	49	F	RR	21	0.0	Teriflunomide	None	Headache	17.7	18.2	4.5	3.610	Yes
5	34	M	RR	1	0.0	Dimethyl fumarate	None	None	20.7	21.0	5.1	1.329	Yes
6	51	F	RR	11	2.0	Teriflunomide	None	Headache	16.8	18.7	5.8	6.568	Yes
7	43	F	RR	31	3.5	Cladribine	4-aminopyridine	None	25.0	25.0	6.4	27.366	Yes
8	45	F	RR	12	2.5	None	None	Overeating	21.5	20.9	5.2	6.646	Yes
9	30	F	RR	5	0.0	Natalizumab	None	None	26.0	23.0	5.2	3.249	No
10	30	F	RR	1	1.0	Cladribine	None	None	22.4	24.8	5.3	13.461	Yes
11	36	M	RR	11	2.5	Dimethyl fumarate	None	None	17.7	19.0	6.4	0.792	Yes
12	32	F	RR	9	1.5	Cladribine	Solifenacin	None	24.5	24.0	6.2	9.930	Yes
13	30	M	RR	3	1.5	None	None	None	22.4	20.0	5.3	7.097	No
14	55	M	RR	28	1.5	None	None	None	23.0	22.4	5.5	20.925	No
15	52	F	RR	9	1.5	Teriflunomide	None	None	18.3	19.5	4.1	12.942	Yes
16	46	F	RR	3	2.5	Peginterfeon beta-1a	None	None	25.0	25.4	6.7	1.522	No
17	48	F	RR	6	1.0	Teriflunomide	None	Depression	16.8	18.9	4.6	0.194	No
18	49	F	RR	7	3.0	Cladribine	Pregabalin	Hypertension	23.5	24.4	7.4	14.331	Yes
19	50	F	RR	5	0.0	Teriflunomide	None	None	18.0	18.9	5.6	0.109	Yes
20	39	M	RR	17	2.0	Fingolimod	None	None	27.8	24.5	8.0	19.155	Yes
21	48	F	RR	14	4.0	Dimethyl fumarate	4-aminopyridine	None	23.8	23.5	6.2	30.400	Yes
22	38	F	RR	4	2.0	Interferon beta-1a	SSRI	Thyroiditis, Obesity, Depression, Arthritis	20.8	20.9	5.9	21.546	Yes
23	40	M	RR	13	4.0	Alemtuzumab	None	Headache	22.1	21.0	5.7	20.694	Yes
24	38	M	RR	8	1.0	Teriflunomide	Tadalafil	None	17.8	17.8	3.9	1.178	Yes
25	23	M	RR	1	0.0	None	None	None	20.7	21.4	5.1	23.227	Yes
26	51	F	RR	8	3.0	None	BZD, Amisulpride	Arthritis	25.5	23.3	5.6	2.418	Yes
27	29	M	RR	6	1.5	Dimethyl fumarate	None	Depression	23.9	20.0	4.2	2.125	Yes
28	43	F	RR	13	2.0	Teriflunomide	None	None	18.5	20.9	4.7	3.730	No
29	48	M	RR	24	4.5	None	SSRI, BDZ	Depression, High blood pressure	20.4	19.0	5.5	9.266	No

30	57	M	RR	33	1.5	None	None	Hyperlipidemia	25.9	28.7	7.7	9.237	Yes
31	43	F	RR	10	2.5	Dimethyl fumarate	Baclofen	ANA positivity	30.0	30.4	8.2	0.670	Yes
32	49	F	RR	11	3.0	Fingolimod	None	High blood pressure	26.0	24.5	12.7	15.768	Yes
33	41	M	RR	19	2.5	Teriflunomide	Solifenacin	Thyroiditis	20.5	21.8	5.5	0.348	Yes
34	47	F	RR	4	4.0	None	SSRI	Endometriosis, Depression	23.9	25.2	6.8	3.276	Yes
35	50	F	RR	16	2.0	Natalizumab	None	None	20.4	20.5	5.8	12.724	Yes
36	42	F	RR	11	1.0	Teriflunomide	None	None	18.2	18.9	5.4	14.256	Yes
37	44	F	RR	7	0.0	None	None	None	21.3	23.5	5.2	8.285	Yes
38	51	M	SP	7	5.0	Fingolimod	Pregabalin	None	22.7	23.5	12.1	7.899	Yes
39	44	M	SP	26	5.0	Fingolimod	Baclofen	None	34.3	42.6	13.7	49.560	Yes
40	47	F	SP	9	4.0	Fingolimod	SSRI	Obesity, Depression	23.5	25.5	7.0	7.068	Yes
41	62	M	SP	14	5.0	Dimethyl fumarate	Tamsulosin	None	29.1	27.4	13.9	14.413	Yes
42	49	F	SP	9	7.0	Siponimod	Baclofen, Tamsulosin, SSRI	Depression	30.2	32.5	N/A	4.148	Yes
43	71	F	SP	33	4.5	Fingolimod	None	High blood pressure	23.3	23.7	7.3	14.356	Yes
44	49	M	SP	26	3.5	Ocrelizumab	SSRI	Depression	24.5	24.4	11.5	23.162	Yes
45	42	F	SP	24	5.5	Ocrelizumab	BoNT, 4-amino-pyridine. BDZ	None	30.8	40.3	10.3	21.894	Yes
46	52	M	SP	18	6.0	Dimethyl fumarate	Delta-9-THC	Depression	61.0	N/A	N/A	36.578	Yes
47	46	F	SP	21	7.5	Ocrelizumab	Delta-9-THC	None	N/A	N/A	N/A	14.500	Yes
48	53	M	SP	3	4.5	Fingolimod	4-aminopyridine	None	25.7	25.5	8.6	29.847	Yes
49	49	F	SP	5	6.0	Fingolimod	Baclofen	None	32.6	34.0	7.4	4.740	Yes
50	42	M	SP	8	3.0	Alemtuzumab	None	None	32.6	28.8	8.1	13.350	Yes
51	59	F	SP	35	2.5	None	Delta-9-THC	None	22.9	23.5	6.4	23.967	Yes
52	53	M	SP	17	5.0	Fingolimod	Baclofen, SSRI, Solifen-acin	Depression	24.8	26.3	8.6	15.703	Yes
53	43	F	SP	6	4.5	Fingolimod	Pregabalin, Solifenacin	Uveitis	23.9	26.7	7.9	21.497	Yes
54	57	M	SP	22	7.0	Ocrelizumab	SNRI, Statins	Depression, Hyperlipidemia	42.0	N/A	N/A	9.324	Yes
55	57	F	SP	24	6.0	Ocrelizumab	SSRI	Depression	33.5	61.0	7.9	8.681	Yes
